# Predictors of risk and resilience to psychopathology in refugee youth: A longitudinal study

**DOI:** 10.1017/S0954579425100576

**Published:** 2025-09-15

**Authors:** Liza M. Hinchey, Rasheed Alahmad, Kathleen Gorski, Mackenzie Jenuwine, Nicole Nugent, David Rosenberg, Tuka Mohiddin, Arash Javanbakht

**Affiliations:** 1 Wayne State University in Detroit, Detroit, MI, USA; 2 Alpert Medical School of Brown University, Providence, RI, USA

**Keywords:** Anxiety, children, posttraumatic stress, refugees, youth

## Abstract

Refugee youth are at high risk for trauma-related disorders – outcomes not only the result of pre-migration trauma, but consequences of diverse post-migration stressors. This study identified individual, parental, and environmental factors – some potentially modifiable – associated with trajectories of psychological risk and resilience in 291 Syrian and Iraqi refugee youth during resettlement in the U.S. Data was collected at arrival and at two follow-up visits up to 7 years post-arrival. Linear mixed modeling assessed predictors of posttraumatic stress disorder (PTSD), anxiety, and depression trajectories. Victimization trauma (i.e., assault) and lower maternal subjective social status predicted more severe PTSD (*p* = .046, *f* ^2^ = .07; *p* < .001, *f* ^2^ = .23) and anxiety (*p* = .008, *f* ^2^ = .05; *p* = .002, *f* ^2^ = .11) trajectories in youth. Paternal unemployment predicted less stable PTSD (*p* = .009, *f* ^2^ = .13) and anxiety (*p* < .001, *f* ^2^ = .10) trajectories. More severe depression trajectories were associated with female sex (*p* = .045, *f* ^2^ = .06) and death threat traumas (*p* = .014, *f* ^2^ = .07). Findings identified predictors of long-term risk and resilience for refugee youth, as well as potentially modifiable ecological risk factors. Victimization and death threat trauma exposure could be salient in identifying youth at high risk for trauma-related symptoms early in resettlement. Indicators of financial security were also associated with symptoms, suggesting environmental intervention targets.

## Introduction

Pre-migration trauma and the substantial stress of post-migration resettlement represent key pillars of mental health risk for refugees (Fazel et al., 2012) – with refugee youth particularly likely to experience long-term impact (Javanbakht et al., 2018). Loss of community and social supports, discrimination, financial and employment insecurity, and social and cultural isolation are among a multitude of chronic stressors faced by displaced families (LeMaster et al., 2018); often, following exposure to traumatic events such as war, assault, torture, or loss of loved ones (Fazel et al., 2012; Hinchey et al., 2024; Hinchey et al. (2023); Javanbakht et al., 2018). Considerably elevated rates of trauma-related symptoms have accordingly been documented in youth resettling as refugees, with up to 42%, 53%, and 33%, meeting diagnostic thresholds for posttraumatic stress disorder (PTSD; Fazel et al., 2005, 2012), anxiety (Javanbakht et al., 2018), and depression (Erol & Seçinti, 2022), respectively. Despite this high prevalence – and the fact that children and adolescents represent 40% of the 43.4 million refugees globally (*United Nations High Commissioner for Refugees (UNHCR): The UN Refugee Agency*, 2024) – refugee youth remain underrepresented in scientific research (Hinchey et al., 2023b). Further, most extant studies focus specifically on PTSD symptoms, whereas anxiety has been less examined, despite representing a more common trauma-related outcome in this population (Javanbakht et al., 2018). Even less is known regarding likely trajectories of trauma- and stress-related symptoms over the course of resettlement, partially as, by definition, the resettlement experience regularly involves tumultuous periods of transition (e.g., of location, contact information), uncertainty (e.g., regarding access to transportation, availability of time/financial resources), and fear (e.g., hesitance to trust authority figures or share personal information; Hinchey et al., 2023b). In short, many of the same factors begetting stress and hardship for refugee families concurrently limit the collection of data on these stressors – data that is essential to providing informed preventative, therapeutic, and social supports.

Despite these constraints, several studies have conducted longitudinal data collection in refugee youth (i.e., children and adolescents), findings from which corroborate the risk of long-term psychological impact. The presence of distinct symptom severity phenotypes over time – including trajectories of stable resilience/low symptoms, chronically elevated symptoms, and delayed symptom onset (Hornfeck et al., 2024) – has been observed, indicating a need to classify predictors and potentially modifiable correlates of risk and resilience. Preliminary findings have indicated the potential for trauma exposure and various resettlement factors to influence symptoms of PTSD, anxiety, and depression over time in youth (Hinchey et al., 2023a; 2023c), though conclusions have been limited by small sample sizes, attrition, and relatively short study duration. Many existing studies note the need for additional research, across longer periods of resettlement, and in multiple cultural contexts (Grasser, 2022; Javanbakht & Grasser, 2022; Ressler et al., 2024). Comparable patterns have been more reliably documented in adult refugee populations (Lenferink et al., 2022), corroborating the salience of understanding these dynamics in refugee youth, for whom much less data is available. Most studies of refugee health are limited to single timepoint, cross-sectional studies – as is the case for the majority of childhood trauma research, wherein most childhood trauma studies are retrospective, while prospective cohort studies are limited (Baldwin et al., 2019; De Bellis & Zisk, 2014). In other words, more is known about adults with histories of childhood trauma, than the impact of trauma on children specifically. Across youth populations, data regarding predictors of symptom change over this sensitive developmental time-period would inform classifications of risk, early intervention, and prevention of chronic or worsening symptoms.

Youth represents multiple periods of high neural and behavioral plasticity – i.e., psychological and neural systems are highly susceptible to both adaptive and maladaptive influence (Grasser & Jovanovic, 2021). This is particularly salient regarding the development of fear- and trauma-related disorders. For one, in healthy adults, regulation of the amygdala and limbic fear response is more developed (Motzkin et al., 2015). Childhood and early adolescence, however, is characterized by a disproportionately more developed amygdala as compared to regulatory brain regions (Silvers et al., 2017), leading to diminished regulation of fear responses in the case of childhood trauma exposure. Additionally, children are particularly receptive to social learning – including learned fear and safety cues (Askew et al., 2016). Given limited cognitive processing capacity, children strongly rely on verbal and nonverbal cues relayed by their trusted adults about safety or threats of their environment. Stress and trauma experienced by parental figures can, therefore, have significant downstream effects on children (Eruyar et al., 2018). Maternal post-migration living difficulties (PMLDs), for instance, have been shown to fully mediate the effect of maternal trauma exposure on youth depression in Syrian refugee families (ElFishawy et al., 2025).

While developmental, family, and neurobiological factors contribute to risk across trauma-exposed youth populations, special considerations also exist for refugee youth. In the context of forced migration and resettlement, studies of trauma in refugee youth (though primarily cross-sectional) have strongly implicated the twofold salience of pre-migration trauma and post-migration resettlement stress. These dynamics are well-explained through an ecological framework (Bronfenbrenner, 1999), which emphasizes the interactions between individual, familial, and environmental systems in shaping development. This lens is particularly well-suited to understanding the multifaceted challenges faced by refugee youth, whose mental health is influenced by pre- and post-migration experiences across multiple ecological levels. Experiences in which one’s life is perceived to be at risk (i.e., “death threat” traumas; Hinchey et al., 2023a) or direct exposure to war (individual level; Hinchey et al., 2023c); parental psychological distress during resettlement, including financial difficulties (family level; Fazel et al., 2012; Masarik et al., 2022); and educational adjustment stress (Masarik et al., 2022), discrimination, or bullying at school (environmental level; DeClercq et al., 2024) have all been associated with negative mental health outcomes in resettling youth. Despite this dual impact, few studies in refugee youth populations have modeled multiple ecological factors simultaneously to characterize overall risk. As many of these factors unfold over time, limited access to longitudinal data has also limited capture of the broad dynamics influencing trajectories of pathology and/or resilience in refugee youth over time. Finally, while there exists robust evidence for adopting a family-oriented approach to investigating refugee youth mental health, few analyses have examined both child and parental factors related to stress and resettlement environment.

The current study aimed to identify individual, parental, and environmental factors – some of which may be modifiable – associated with trajectories of mental health risk and resilience (herein operationalized as lower symptom severity/lack of symptoms) in a sample of Syrian and Iraqi refugee youth during the years following resettlement in the U.S. We sought to strengthen extant literature via a longitudinal study design, spanning approximately seven years post-migration; a focus on multiple domains of major psychosocial influence (e.g., individual trauma exposure, parental PMLDs, socioeconomic status); and investigation of PTSD, anxiety, and depression as key trauma- and stress-related outcomes. Contractor et al.’s (2020) taxonomy of trauma subtypes was employed, given evidence across multiple populations (Arnetz et al., 2014) – as well as in the current cohort (Hinchey et al., 2023; Hinchey et al., 2023) – for the improved predictive power of stratifying trauma exposure by subtype (e.g, assault-based trauma vs. accident-related trauma), rather than querying cumulative trauma (i.e., total number of exposures endorsed on a trauma screener). Guided by an ecological framework and existing empirical findings, we focused on individual trauma exposure, parental post-migration stress, and indicators of environmental stability to inform variable selection and hypotheses. We hypothesized that: 1) youth exposed to victimization and death threat trauma would exhibit worse symptom trajectories; 2) more severe resettlement stress reported by parents (as defined by PMLDs and perception of one’s environment quality) would be associated with less favorable outcomes for youth; and 3) markers of higher socioeconomic standing (e.g., paternal employment, parental subjective social status) would predict more resilient youth symptom trajectories.

## Methods

### Participants and procedures

In 2016, recruitment of a cohort of accompanied Syrian and Iraqi youth resettling as refugees began in southeast Michigan (*n* = 291). Recruitment occurred at primary care clinics providing mandatory health screenings during the first month of arrival; after screening, medical providers shared information about the study and introduced interested families to the research team on site. Bilingual (Arabic- and English-speaking) research assistants completed informed consent and assent processes and administered surveys in a private room (87.7% of families attending health screenings participated). For a detailed report of participants and procedures, see prior work (Javanbakht et al., 2019, 2020). Following this initial data collection (*n* = 178), participants provided data at two more time points, approximately 2 to 3- and 4 to 5-years post-arrival in the U.S. (*n* = 81 and *n* = 40, respectively). To account for attrition – a common barrier to longitudinal refugee research due to the realities of resettlement (Hinchey et al., 2023b) – additional participants (resettled for approximately 4 to 5 years) were recruited and interviewed during the second wave of data collection (*n* = 113); follow up was conducted with this group approximately 7 years post-resettlement (*n* = 97). As is the norm culturally, all youth remained living in the parental home for the duration of the study. Inclusion criterion for both cohorts were: (1) Syrian or Iraqi refugee youth between the ages of 6 and 17, (2) accompanied by at least one parent. Exclusion criteria were as follows: (1) unaccompanied minors, (2) wardens of the court, and (3) past or current psychosis diagnosis. All study procedures were carried out in accordance with the latest version of the Declaration of Helsinki and approved by the Institutional Review Board at Wayne State University – IRB #012416B3F.

### Measures

Self-report measures were administered by culturally and linguistically fluent research staff and were made available in both Arabic and English. Arabic questionnaires were translated from English versions by a native speaker, back translated by a different Arabic speaker to ensure accuracy, and approved by external review. Sample characteristics were obtained using a demographic questionnaire. For this analysis, all measures listed under ***Adult Measures*** were only completed by adults and all those under ***Youth Measures*** were only completed by youth.

#### Adult measures

Parental survey data from the second time point were used to capture accurate assessments of lifestyle (measures detailed below), as compared to upon arrival. Due to participant hesitance towards sharing income levels (often derived from fears of losing social benefits; Hinchey et al., 2023b), the MacArthur Scale of Subjective Social Status (MacArthur SSS) was used to operationalize subjective socioeconomic status (Operario et al., 2004). Respondents rank their social status by placing an “X” on two visuals of ladders. Ladder 1 prompts participants to place an “X” on the rung they feel they stand on compared to their own community; Ladder 2, compared to other people in the U.S. Scores range from 1 to 10, with higher values indicating higher SSS.

An adapted version of the Post-Migration Living Difficulties checklist (PMLD) was administered to parents to assess degree of exposure to common post-migratory stressors (e.g., isolation/separation from community, financial/employment challenges, access to health services, etc.). The PMLD was originally generated in direct conversation with refugee and immigrant communities (Silove et al., 2017) and transcultural validity has since been established (Alemi et al., 2016). Respondents rate the extent to which each stressor is a problem for them; items are then summed to comprise a composite score. Cronbach’s alpha indicated excellent reliability (*α*=.905).

The 62-item Perceived Residential Environment Quality Indicators (PREQIs) measure was used to operationalize perception of resettlement and neighborhood quality in parents. The PREQIs prompts respondents to indicate level of agreement with statements related to the quality of public transport, neighborhood upkeep, social services, community attitudes and friendliness, and other environmental components (Bonaiuto et al., 1999). Item scores are summed, with higher composite scores indicating better environment quality. The PREQIs has demonstrated validity and reliability across cultures and contexts (Bonaiuto et al., 2015), and was highly reliable in the current sample (*α*=.899).

#### Youth measures

The Life Events Checklist of the DSM-5 (LEC 5) was used to query trauma exposure. The LEC 5 prompts respondents to indicate level of exposure to 16 potentially traumatic events, and is valid and reliable across populations and cultures (Rzeszutek et al., 2018). “Yes” responses to having directly experienced an event (i.e., “Happened to me”) were summed to calculate continuous scores within each trauma subtype, as defined by Contractor et al. (2020). Cumulative trauma scores were also calculated, to investigate best model fit during main analyses.

The 31-item UCLA PTSD Reaction Index for Children and Adolescents (UCLA PTSD RI) for DSM 5 assessed severity of PTSD-related symptoms. The UCLA PTSD RI prompts participants to indicate the extent to which they have been bothered by various symptoms over the past month; scale-level symptom severity scores are then calculated by summing all items (Steinberg et al., 2013). The UCLA PTSD RI is valid and reliable across cultures, and is specifically recommended for refugee youth populations (Javanbakht et al., 2018). Cronbach’s alpha evidenced excellent reliability in this sample (*α*=.928).

Anxiety symptom severity was assessed using the 41-item Screen for Child Anxiety-Related Emotional Disorders (SCARED; Birmaher et al., 1997), which asks participants to report the level to which each item describes their experiences (not true: 0; often true: 1; very true; 2); item scores are then summed to comprise a scale score. The SCARED has been cross-culturally validated, is reliable in longitudinal designs following children to young adulthood, and is valid in refugee populations (Hale et al., 2008; Javanbakht et al., 2020). Internal reliability in this sample was excellent (α=.925).

Depression symptom severity was evaluated via the 33-item Mood and Feelings Questionnaire (MFQ). The MFQ was designed specifically for youth populations and solicits the extent to which specific depression symptom items are true for participants (Costello & Angold, 1988). The scale is valid and reliable across multiple cultures and populations (Kim et al., 2021), and Cronbach’s alpha indicated excellent reliability in the current sample (*α*=.931).

### Statistical analyses

Analyses were conducted in SPSS version 29.0 and figures produced using the “ggplot2” package in R version 1.4.1106. Distributional properties, missing data, and adherence to model assumptions were assessed using descriptive statistics. Person-median responses on scale measures were used to impute missing item-level data when 40% or fewer responses were missing on the UCLA PTSD RI, SCARED, MFQ, PMLD, and PREQIS (Newman, 2014); surveys with more items missing were considered missing at scale level. Univariate outliers were identified by median absolute deviation (MAD) and corrected using winsorization (Khalil et al., 2024). A significant Kolmogorov-Smirnov test and histogram indicated positive skew of UCLA PTSD RI sum scores; thus, this variable was log-transformed. Data screening yielded no other deviations from normality. Pairwise plots were evaluated to assure linearity, homoskedasticity, and normality of residuals; no assumptions were violated. For all analyses, Cook’s distance confirmed there were no disproportionately influential data points (Zhu et al., 2012). Pairwise correlations revealed no issues of multicollinearity (*r* < .9 for all), and, alongside t-tests, were inspected to identify necessary covariates for each outcome variable. A two-tailed *t*-test indicated a significant sex difference in anxiety severity (*p* > .001). No other demographic candidate controls were identified (using *r* > .3 as cut off); age and sex were included as covariates in all subsequent analyses based on prior literature (Breslau, 2002). Percentages of missing data for analysis variables were as follows: 0% child demographic variables; < 5% trauma exposure; < 45% anxiety and PTSD (Time 1); and 68.9% depression (Time 1); < 10% symptom scales (Time 2 and 3); < 50% parental employment (Time 2); < 27% for parental scale variables (Time 2). Data was found to be missing completely at random (MCAR; Little’s x^2^ = 90.72). Given high levels of missing depression data at Time 1, depression models examined trajectories from Times 2 and 3 during resettlement only.

To examine potential predictors of risk and resilience to symptoms, time-unstructured linear mixed modeling (LMM) was performed. LMM is a robust statistical technique for autoregressive data that accounts for baseline inter-individual symptom variation; simultaneously examines predictors of symptom variation over time; and handles missing and unbalanced longitudinal data well using maximum likelihood estimation (Singer & Willett, 2003). As time-unstructured models offer more precise estimates when intervals between data collection time points are not consistent across individuals, time was operationalized as number of days since arrival in the U.S. Three models were developed to investigate PTSD, anxiety, and depression symptom trajectories. Models were built with a nested approach, using likelihood ratio testing, as well as assessment of p-values and pseudo-R^2^ values, to obtain best fit and a parsimonious model(Singer & Willett, 2003); see Tables S.1, S.2, and S.3. Given the autocorrelation of residuals across time points in longitudinal data, an autoregressive covariance structure (AR1) was specified (Singer & Willett, 2003). For all models, time, age, sex, trauma exposure variables, and environmental factors reported by mothers and fathers were entered as fixed effects. To examine interaction effects of time and predictors on symptom severity, interaction vectors were included as fixed effects as follows:

#### Time X predictor variable

Allowing for random slopes did not improve model fit; therefore, only intercepts were entered as random effects. Marginal and conditional pseudo-R^2^ values provided estimates of variance explained by final models, as described by Nakagawa S, & Schielzeth (2013).

## Results

### Descriptive statistics

Sample characteristics are presented in Table 1. Of the 291 youth included across both recruitment cohorts, sample size across the three time points was reasonably stable (*n* = 177, *n* = 195, and *n* = 139, respectively). Mean age of participants at time of arrival (baseline) was 11.66 (SD = 3.16; range = 6 to 17) and approximately half were female (47.0%). The cohort was 77.8% Syrian and 22.2% Iraqi. 88.7% reported Arab ethnicity, 9.1% Chaldean/Asyrian, and 2.2% Kurdish. 41.2% of participants’ mothers (*n* = 104) and 73.9% of fathers (*n* = 91) were employed by the first follow-up visit. Average number of cumulative trauma exposures endorsed by youth was .95 (SD = 1.75; range = 0 to 14), with 17, 29%, and 42% reporting victimization, accident/injury, and death threat trauma, respectively. Across resettlement, average depression *(M* = 12.53, SD = 12.01) and PTSD *(M* = 21.23, SD = 20.58) symptom severity was low-to-moderate, whereas anxiety symptom severity was more severe, with average anxiety *(M* = 25.95, SD = 14.9) reaching the diagnostic cut-off for a probable anxiety disorder ( ≥ 25).(Birmaher et al., 1997)

**Table 1.** Demographic characteristics and descriptive statistics of full sample at each time point (n = 291)

Variable	Time 1 (*n* = 177)			Time 2 (*n* = 195)			Time 3 (*n* = 139)		
	Range	*M*	SD	Range	*M*	SD	Range	*M*	SD
Age (at arrival in U.S.)	6 – 17	11.66	3.16	7 – 20	13.05	3.20	9 – 22	14.94	3.22
Female, %		44.0			47.0			50.4	
Country of origin, %									
Syria		71.6			94.8			98.9	
Iraq		28.4			5.2			1.1	
Ethnicity, %									
Arab		85.3			97.8			100.0	
Kurdish		2.8			1.5			0.0	
Chaldean/Asyrian		11.9			.7			0.0	
Maternal education, %									
Illiterate		8.2			3.8			6.3	
Elementary school		39.8			55.7			61.9	
High school		39.8			26.4			19.0	
College/University		12.3			14.2			12.7	
Higher education		0.0			0.0			0.0	
Paternal education, %									
Illiterate		3.6			6.2			1.7	
Elementary school		35.5			42.3			50.0	
High school		53.6			48.5			44.8	
College/University		7.2			3.1			3.4	
Higher education		0.0			0.0			0.0	
Mother employed, %		–			19.2			10.6	
Father employed, %		–			66.7			75.6	
Maternal SSS 1	1 – 8	5.07	2.22	1 – 9	5.13	2.08	1 – 9	5.05	1.96
Maternal SSS 2	1 – 9	4.86	2.16	1 – 9	4.80	2.04	1 – 8	4.48	1.95
Paternal SSS 1	1 – 8	4.65	2.15	1 – 8	4.69	1.98	1 – 8	4.79	1.98
Paternal SSS 2	1 – 9	4.39	2.04	1 – 9	4.39	2.11	1 – 8	4.48	2.02
Maternal LDQ	2 – 61	24.74	15.35	1 – 61	20.98	13.95	2 – 47	20.62	13.21
Paternal LDQ	1 – 69.5	26.17	21.03	1 – 69.5	22.06	18.96	1 – 69.5	20.05	18.33
Maternal PREQIs	16.3 – 63.5	51.28	13.76	16.3 – 63.7	50.86	11.29	16.25 – 63.65	50.63	11.32
Paternal PREQIs	17.6 – 62.3	49.86	15.26	17.6 – 63.7	49.91	12.95	22.75 – 63.65	51.93	10.28
Accident/Injury	0 – 4	.29	.60	0 – 4	.27	.53	0 – 3	.27	.54
Victimization	0 – 3	.17	.49	0 – 3	.17	.48	0 – 3	.17	.48
Death threat	0 – 6	.42	.96	0 – 6	.41	.93	0 – 5	.41	.93
Cumulative trauma	0 – 14	.95	1.75	0 – 14	.94	1.63	0 – 11	.94	1.63
Anxiety Sxs	0 – 57	23.61	13.04	0 – 57	24.53	14.88	0 – 57	24.31	14.92
PTSD Sxs	0 – 80	17.84	18.11	0 – 98	21.97	20.50	0 – 91	19.83	18.43
Depression Sxs	0 – 65	11.32	11.97	0 – 62	11.71	11.11	0 – 55	11.24	10.93

*Note.* Data from Time 2 was used for several parental variables (i.e., employment, LDQ, SSS 1 and 2, and PREQIs) in analyses to capture realistic assessment of lifestyle, as opposed to directly upon arrival to the U.S. Blank cells indicate that data was not collected for this variable during the relevant time point. Ranges refer to the range of sample, not possible range of scores on measures. LEC-5 items included in each subtype category are as follows: 1) accident/injury (items 1 – 4 and 12); 2) victimization (items 6, 8, and 9); and 3) death threat (items 5, 7, 10, 11, and 13 – 16; Contractor, 2020). Abbreviations: Symptoms (Sxs); Subjective Social Status 1 (SSS 1); Subjective Social Status 2 (SSS 2); Living Difficulties Questionnaire (LDQ); Perceived Residential Environment Quality Indicators (PREQIs).

### PTSD trajectories

**Figure 1.**
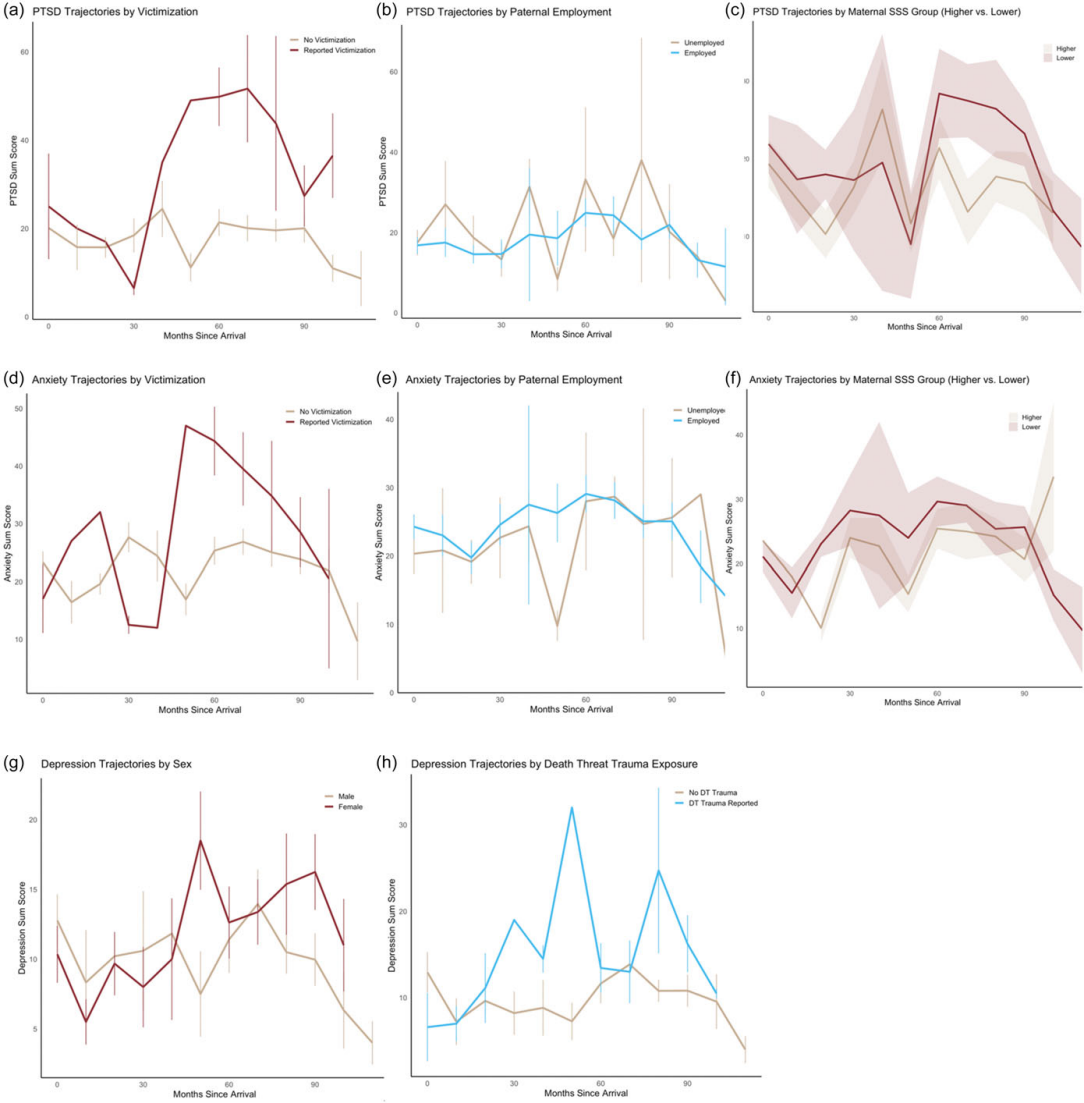
Trajectories of PTSD symptoms by (a) victimization trauma exposure, (b) paternal employment status, and (c) maternal subjective social status; anxiety symptoms by (d) victimization trauma exposure, (e) paternal employment status, and (f) maternal subjective social status; and depression symptoms by (g) sex, and (h) death threat trauma exposure.

Based on model building and best-fit procedures described above, a random-intercepts fixed-slopes LMM model was developed to estimate predictors of PTSD symptom trajectories (Table 2; Figure 1). The model revealed significant interaction effects of *Time X Victimization* (*b* = .01, *t* = 2.06, *p* = .046, *f* ^*2*^ = .07), *Time X Employment (paternal)* (*b* = .01, *t* = 2.74, *p* = .009, *f* ^*2*^ = .13), and *Time X Subjective Social Status 1 (maternal)* (*b* = −.00, *t* = -3.60, *p* < .001, *f* ^*2*^ = .23). Marginal *R* ^*2*^ indicated that 49.2% of the variance in PTSD severity was explained by fixed effects; fixed and random effects explained 74.6% of the variance (conditional *R* ^*2*^).

**Table 2.** Results of final linear mixed-effects models predicting child PTSD symptom severity

Variable	Est/Beta	SE	95% CI	*t*	*p*	f^2^
Intercept	5.44	2.90	−.42 – 11.29	1.87	.068	
Time	−.00	.02	−.05 – −.04	−.09	.928	
Age	.00	.00	−.00 – .00	1.43	.161	
Female	.72	2.55	−4.44 – 5.87	.28	.780	
Victimization	−6.27	4.47	−15.30 – 2.75	−1.40	.168	
Accidental/Injury	2.07	1.15	−.25 – 4.38	1.81	.078	
Death threat	2.35	2.51	−2.71 – 7.42	.938	.354	
Employment (paternal, Yes = 1)	−.76	2.73	−6.27 – 4.75	−.28	.782	
MacArthur SSS1 (maternal)	1.87^***^	.44	.98 – 2.76	4.26	< .001	
LDQ (maternal)	−.20^*^	.08	−.37 – −.03	−2.41	.020	
Time X age	5.49^-7^	6.64^-6^	−.00 – −.00	.08	.934	
Time X victimization	.01^*^	.00	−.00 – .01	2.06	.046	.07
Time X death threat	−.00	.00	−.00 – .00	−.89	.376	
Time X employment (paternal, Yes = 1)	.01^**^	.00	.00 – .01	2.74	.009	.13
Time X LDQ (maternal)	8.97^-5^	4.77^-5^	−6.66^-6^ – .00	1.88	.067	
Time X MacArthur SSS1 (maternal)	−.00^***^	.00	−.00 – −.00	−3.60	< .001	.23
Random Effects						
				Variance	SE	
Residual				8.06	2.69	
Intercept (subject)				8.06	2.99	
Model fit						
Cumulative Trauma Model						
Pseudo-R^2^			Marginal	Conditional		
			0.492	0.746		

*Note.* Time is operationalized as days since arrival in the U.S. Estimates reflect log transformed UCLA PTSD RI scores, multiplied by 10 to maintain consistency of scaling. Cohen’s f^2^ was calculated using marginal R^2^ values for significant associations of interest. Abbreviations: Random effects (RE); Fixed effects (FE); Living Difficulties Questionnaire (LDQ); MacArthur Subjective Social Status 1 (MacArthur SSS1). Pseudo-R^2^ values were calculated using SPSS version 29.0 software via methods described in Nakagawa & Schielzeth (2013).

* *p* < .05

** *p* < .01

*** *p* < .001.

### Anxiety trajectories

A random-intercepts fixed-slopes LMM model was built to query predictors of anxiety symptom trajectories during resettlement (Table 3; Figure 1). Among variables of interest, significant interaction effects of *Time X Victimization* (*b* = −.02, *t* = −2.74, *p* = .008, *f* ^*2*^ = .05), *Time X Employment (paternal)* (*b* = −.01, *t* = −3.73, *p* < .001, *f* ^*2*^ = .10), and *Time X Subjective Social Status 1 (maternal)* (*b* = .00, *t* = 3.22, *p* = .002, *f* ^*2*^ = .11) emerged. Pseudo *R* ^*2*^ values indicated that fixed effects explained 45.9% of the variance in anxiety symptom severity, while fixed and random effected explained 59.0%.

**Table 3.** Results of final linear mixed-effects models predicting child anxiety symptom severity

Variable	Est/Beta	SE	95% CI	*t*	*p*	f^2^
Intercept	107.39	26.31	55.05 – 159.73	4.08	< .001	
Time	−.03^*^	.01	−.06 – −.01	−2.41	.018	
Age	.36	.75	−1.13 – 1.84	.48	.633	
Female	.46	4.70	−8.90 – 9.82	.10	.922	
Victimization	52.64^**^	18.38	15.94 – 89.34	2.86	.006	
Death threat	−5.62	7.85	−21.23 – 9.99	−.72	.476	
Employment (paternal, Yes = 1)	20.89^***^	5.44	10.07 – 31.71	3.84	< .001	
PREQIS (maternal)	−1.34^*^	.67	−2.67 – −.01	−2.01	.048	
MacArthur SSS1 (maternal)	−5.17^***^	1.32	−7.81 – −2.53	−3.90	< .001	
PREQIS (paternal)	−.10	.48	−1.06 – .86	−.21	.837	
Time X age	−.00^**^	.00	−.00 – .00	−2.86	.005	.11
Time X female	.00	.00	−.00 – .01	1.44	.154	
Time X victimization	−.02^**^	.01	−.04 – −.01	−2.74	.008	.05
Time X death threat	.01	.00	−.00 – .01	1.57	.120	
Time X employment (paternal, Yes = 1)	−.01^***^	.00	−.02 – −.01	−3.73	< .001	.10
Time X PREQIS (maternal)	.00	.00	.00 – .00	1.47	.146	
Time X MacArthur SSS1 (maternal)	.00^**^	.00	.00 – .00	3.22	.002	.11
Time X PREQIS (paternal)	.00	.00	.00 – .00	1.56	.122	
Random effects						
				Variance	SE	
Residual				88.27	19.33	
Intercept (subject)				28.15	20.23	
Model fit						
Cumulative Trauma Model						
Pseudo-R^2^			Marginal	Conditional		
			0.459	0.590		

*Note.* Time is operationalized as days since arrival in the U.S. Cohen’s f^2^ was calculated using marginal R^2^ values for significant associations of interest. Abbreviations: Random effects (RE); Fixed effects (FE); Living Difficulties Questionnaire (LDQ); Perceived Residential Environment Quality Indicators (PREQIs); MacArthur Subjective Social Status 1 (MacArthur SSS1). Pseudo-R^2^ values were calculated using SPSS version 29.0 software via methods described in Nakagawa & Schielzeth (2013).

* *p* < .05

** *p* < .01

*** *p* < .001.

### Depression trajectories

Given the high proportion of missing MFQ data at time of arrival (68.9%), only data from the two follow-up time points were examined. All other model building procedures and specifications were identical to the previous LMMs. Interaction effected were observed for *Time X Female* (*b* = .01, *t* = 2.05, *p* = .045, *f* ^*2*^ = .06) and *Time X Death Threat* (*b* = .01, *t* = −2.55, *p* = .014, *f* ^*2*^ = .07; Table 4; Figure 1). Marginal *R* ^*2*^ suggested that 32.5%% of the variance in depression symptom severity was explained by fixed effects, while conditional *R* ^*2*^ indicated that fixed and random effects explained 58.1% of the outcome variance. Due to the significant sex effect, post-hoc analysis was conducted on sex-disaggregated data. Findings indicated that the *Time X Death Threat* relation may be driven by female participants (Female: *b* = .015, *t* = 3.03, *p* = .007; Male: *b* = .003, *t* = .80, *p* = .436) and that *Time X Victimization* was also significant in females (*b* = −.02, *t* = −2.24, *p* = .036); Model convergence, however, was not achieved in the male sample – likely due to sample size – limiting reliability of sex-disaggregated findings.

**Table 4.** Results of final linear mixed-effects models predicting child depression symptom severity

Variable	Est/Beta	SE	95% CI	*t*	*p*	f^2^
Intercept	20.82	12.82	−4.84 – 46.48	1.62	.110	
Time	.01	.01	−.01 – .02	.992	.325	
Age	.06	.75	−1.44 – 1.56	.083	.934	
Female	−8.49	5.16	−18.81 – 1.83	−1.65	.105	
Victimization	24.30	13.49	−2.86 – 51.46	1.80	.078	
Death threat	−14.43^*^	6.44	−27.34 – −1.52	−2.24	.029	
MacArthur SSS1 (paternal)	−.73	.58	−1.90 – .43	−1.27	.212	
PREQIS (paternal)	−.10	.14	−.38 – .18	−.75	.458	
Time X age	−.00	.00	−.00 – .00	−1.21	.233	
Time X female	.01^*^	.00	−.00 – .01	2.05	.045	.06
Time X victimization	−.01	.01	−.02 – .00	−1.51	.139	
Time X death threat	.01^*^	.00	−.00 – .01	2.55	.014	.07
Random effects						
				Variance	SE	
Residual				36.882	8.93	
Intercept (subject)				22.42	10.56	
Model fit						
Cumulative trauma model						
Pseudo-R^2^			Marginal	Conditional		
			0.325	0.581		

*Note.* Time is operationalized as days since arrival in the U.S. Depression model reflects Phases 2 and 3 of data collection (approximately 2- and 3-years post-arrival); depression data was inconsistently collected during Phase 1 (i.e., time of arrival) and impacted model stability. Sensitivity analyses including Phase 1 were also completed, with Time X PREQIS (paternal) association surviving sensitivity analysis. Cohen’s f^2^ was calculated using marginal R^2^ values for significant associations of interest. Abbreviations: Random effects (RE); Fixed effects (FE); Perceived Residential Environment Quality Indicators (PREQIs); MacArthur Subjective Social Status (MacArthur SSS1). Pseudo-R^2^ values were calculated using SPSS version 29.0 software via methods described in Nakagawa & Schielzeth (2013).

^*^p < .05

^**^p < .01

^***^p < .001

## Discussion

This longitudinal study applied an ecological framework to investigate individual, family, and environmental factors associated with trajectories of trauma-related symptoms in refugee youth resettling in the U.S. Regarding PTSD and anxiety symptom trajectories, our first and third hypotheses were supported. Specifically, victimization trauma exposure was associated with more severe and chronic symptom trajectories over the study period (small-to-medium effects); lower maternal subjective socioeconomic status was also associated with more severe symptom trajectories (medium effect); and paternal unemployment was linked to greater instability in symptom trajectories (small-to-medium effects). Depression models also supported our first hypothesis, as those exposed to death threat trauma experienced more severe and chronic depression symptoms over time (small-to-medium effect). Our second hypothesis was not supported, as PMLDs and environment quality did not emerge as significant predictors of symptom change over time – though PMLDs have been shown to impact symptoms in cross-sectional studies (Gleeson et al., 2020).

Here, and in previous cross-sectional and trajectory studies in adults and youth (Hinchey et al., 2023a, 2023c; Radtke et al., 2024), victimization and death threat trauma (individual level factors) have consistently emerged as significant contributors to worse symptom outcomes, collectively signifying the importance of moving away from an approach solely based on summing number of traumatic events experienced, and toward a more in-depth understanding of the severity and contextual aspects of traumatic experiences. These findings also suggest that distinct neurobiological underpinnings may explain the impact of interpersonal trauma types (e.g., victimization and some death threat traumas) compared to more general and less human interaction based traumatic experiences (e.g., non-interpersonal traumas, such as accident/injury traumas). As a social species, we appear to experience more negative impact after traumas inflicted directly by other humans, particularly in their intentionality (Contractor et al., 2020). The unique meaning of these experiences and their social context play an important role in their long-term impact, potentially through both severity of trauma and the influence they have on the cognitive constructs an individual develops regarding the traumatic experiences and themselves (e.g. PTSD criterion D; Boals, 2018) .

Understanding the differential impact of traumatic experiences on longitudinal course of symptoms also has clinical significance. Specifically, given limited resources available for medical care for refugees and war-exposed populations (Hinchey et al., 2023b), as well as lower income populations in general, gaining insight into the specific experiences that predict a more severe course of symptoms could guide resources in prioritizing screening, care, and focused attention in regular medical and mental health visits.

Our findings also demonstrate that maternal perception of family income and paternal objective measure of employment (family/environment level factors) are both salient in predicting resilience among youth. Notably, these findings shed light on potentially modifiable socioeconomic and environmental variables that could mitigate the long-term impact of war trauma and resettlement stress on children – particularly if interventions are early or prevention-based. The impact of maternal perception of socioeconomic status suggests a stronger connection between children’s mental health and their mother’s perception of environmental and financial stress, as children may be more attuned and connected with mother’s stress as the main source of emotional support within the family, as compared to the father’s (Gleeson et al., 2020). Within this population, fathers are often expected to be highly engaged with work to be able to provide for the family. Reduced presence of fathers in regular family interactions and more time spent between youth and their mothers may signify greater emotional attunement between the child and the mother (Malmberg & Flouri, 2011). It is also possible that mothers who worry about finances more likely have children also prone to worry (i.e., a reciprocal mother/child dynamic).

Additionally, paternal employment as an objective indicator of the family’s access to financial support, also contributed to their children’s long-term resilience. This is not only an objective indicator of the family’s more favorable access to resources but could also indirectly indicate the father’s favorable mental health and ability to navigate the host environment effectively. Adult data from Syrian refugees upon arrival in the U.S. indicated high prevalence of PTSD (32.2%), anxiety (40.3%), and depression (47.7%; Javanbakht et al., 2019), all of which detract from work productivity (Drake & Wallach, 2020; Gleeson et al., 2020). In addition to the bidirectional relationship between employment and job satisfaction with psychological functioning, employment status may enhance language skills, social connectedness, and/or supports within the new environment which may be expected to impact the family wellbeing. Alternatively, lack of employment has its own negative impact on the father’s mental health and that of the family (Drake & Wallach, 2020). Overall, a focus on programming that promotes employment and improves the financial status of refugee families appears salient regarding the long-term mitigation of mental health stress in refugee youth – and is indeed economically cost-effective via the reduction of long-term disability and healthcare costs among these individuals (Drake & Wallach, 2020). Childhood poverty not only has negative effects on mental health outcomes and neurodevelopment in childhood, but is also related to adverse mental health outcomes in emerging adulthood, regardless of adult socioeconomic status (SES; Evans & Cassells, 2014). More generous anti-poverty programs may decrease the likelihood of youth experiencing stressors associated with low SES, thereby protecting against the adverse effects on brain development and mental heal (Weissman et al., 2023).

Interestingly, more detailed measures of environmental satisfaction (PMLD and PREQIs) did not correlate with symptom trajectories. This does not necessarily mean that these variables are unimportant, as cross-sectional data have demonstrated their utility in assessing resettlement environmental stress in youth’s mental health (Gleeson et al., 2020). In other words, these factors may not best predict long-term changes but instead have more immediate impact on youth. Alternatively, as refugee families often receive more homogenous initial support early in their resettlement, it is possible that less variance among these variables led to lack of significance in predicting long term mental health symptoms. Notably, PMLD and PREQIs are more complex questionnaires and require more time to complete, while maternal perception of SES and paternal employment are much easier and faster to capture – especially in busy clinical settings.

Together, these findings again signify the importance of moving away from traditional approaches conceptualizing trauma as a static construct by investigating its impact cross-sectionally. Traditionally, studies assess an individual at some point in the journey after their trauma exposure, considering those who exceed a certain DSM-based diagnostic threshold as “disordered,” and others (perhaps even one score lower on symptom measures) as “resilient.” Clinical experiences and data like that assessed herein, however, reveal significantly more complex ecological dynamics among trauma, time, and psychosocial and environmental stress in the evolution of symptoms, particularly for populations with complex and volatile environmental circumstances. An ecological approach is particularly important during the plastic developmental stage of trauma-exposed youth. Evidence-informed approaches to addressing symptom evolution suggest regular screening for trauma outcomes (e.g., PTSD, anxiety, depression) within primary care settings via brief screeners during annual or other healthcare visits, as trauma may not only reoccur, but changes in environmental stress might impact course of symptoms and provoke distress during a sensitive developmental time. This is especially important for those exposed to victimization or death threat trauma experiences. Early capture of high risk for severe symptom trajectories – often leading to impairment in academic, social, and personal life domains – could mitigate the long-term impact of trauma on biopsychosocial health prosperity of refugees.

Another notable finding was a difference in depression symptom trajectories among boys and girls – a difference that did not emerge regarding PTSD and anxiety. While there is cumulative data on sex-effects in anxiety, depression, and trauma-related disorders in adults, indicating a reliably larger prevalence and impact among females (Abu Suhaiban et al., 2019), less is known about sex differences during pre- and peri-pubertal age. While similar sex-effects in depression have been documented in adults, the lack of sex-effects herein regarding anxiety and PTSD may suggest lack of homogeneity in trauma type exposure among girls and boys. Previous findings of higher prevalence of more PTSD in boys than girls upon arrival in the U.S. may support this hypothesis (Javanbakht et al., 2018). Overall, findings support expansion of research into possible sex-effects regarding trauma exposure, from its biological impact to psychosocial aspects and the cultural meaning of such experiences in youth.

## Limitations

This study has strengths and weaknesses. Most participants were recruited upon arrival in the host community, providing an opportunity to better capture the impact of new environmental stressors. A phased recruitment strategy was leveraged to mitigate the impact of high attrition, a common limitation in refugee research. Though LMM handles such designs well, conclusions would have been strengthened by following the same participants across the full study period. Several factors enhance generalizability in refugee populations, including near 90% recruitment success rate, and diversity in ethnicity and country of origin. Still, it is essential to avoid oversimplifying refugee experiences by assuming uniform impact across populations with varied countries of origin, war exposures, and host communities. For instance, Southeast Michigan is home to the largest Arab American community in the U.S., which may provide greater social support and resources for acculturation in contrast to other host communities. A common challenge in refugee populations (Hinchey et al., 2023b), percentages of missing data on several variables were also moderately high (see **Statistical Analyses** in Methods and **Percent Missing Data by Phase** in supplementary materials). Though LMM handles missing data well, models built were limited in their complexity, as high levels of missing data occasionally caused convergence issues during model building. Possibly, variables like parental PMLD and PREQIs – which have been shown to impact youth mental health in cross-sectional analyses – were limited in their utility due to missing data percentages. Additionally, findings regarding depression symptoms in youth apply only to 2- to 7-years post-resettlement, as high missingness (68.9%) in depression data upon arrival precluded its inclusion in analyses. Finally, we acknowledge that defining resilience solely as the absence of psychopathology – as done herein based on available data – is a limited approach that may not capture the full complexity of adaptive functioning following adversity.

## Conclusion

Our findings highlight the importance of ecological, longitudinal approaches to investigating the impact of trauma, forced migration, and environmental stress in youth, both in research and clinical settings. Attention to the differential impact of specific trauma types can guide future studies of their potentially unique neurobiological and cognitive impact, as well as focused clinical screening and follow up. This work also suggests employment and family financial resources as potentially modifiable variables that could mitigate the long-term impact of trauma and environmental stress on our future adults.

## Supporting information

10.1017/S0954579425100576.sm001 Hinchey et al. supplementary material Hinchey et al. supplementary material

## Data Availability

Wayne State University requires data use agreements to be drafted and approved prior to any data sharing. If investigators are interested in accessing data, agreements will need to be drafted and approved between institutions and investigators in order to protect these data. Therefore, data cannot be made publicly available at this time, however, data can be made available by request to the authors and institutional approval with an appropriate data use agreement.

## Supplementary material

The supplementary material for this article can be found at https://doi.org/10.1017/S0954579425100576.
