# Supplementary material for: Predictors of risk and resilience to psychopathology in refugee youth: A longitudinal study
Source: Dev Psychopathol. 2025 Sep 15:1–12. Online ahead of print. doi: 10.1017/S0954579425100576 (PMC12752476; doi:10.1017/S0954579425100576)
Supplement: Hinchey et al. supplementary material [file S0954579425100576sup001.docx]

**Table S.1**

*Model comparison and model building process for PTSD*

| Sampling Units | | N total observations = 420  N subjects = 291 | | | | | | | | | |
| --- | --- | --- | --- | --- | --- | --- | --- | --- | --- | --- | --- |
|  | | | | | | | | | | | |
| Model Specification | **Model Name** | **Nested Model** | **Fixed Effects** |  | **Random Effects** | **Model fit** | | | | **LRT Test against nested** | |
|  |  |  |  |  |  | **AIC** | **BIC** | **-2 LL** | **df** | **df** | **X^2^** |
| RE only | Null | - | - |  | intercepts | 570.5 | 582.6 | 564.5 | 3 | - | - |
| FE main effect | Model 1 | Null | Time |  | intercepts | 566.0 | 582.2 | 558.0 | 4 | 1 | 6.5* |
| FE two-way interactions; controls | Model 2 | Model 1 | Time + Age + Sex + Victimization + Death Threat + Accident/Injury + AgeXTime + SexXTime + VictimizationXTime + Death ThreatXTime + Accident/InjuryXTime |  | intercepts | 471.3 510.2 451.3 10 | | | | 6 | 106.7* |
| FE two-way interactions | Model 3 | Model 1 | Time + Age + Sex + Victimization + Death Threat + Accident/Injury + PEmployment + MLDQ + MMSSS1 + AgeXTime + SexXTime + Accident/InjuryXTime + VictimizationXTime + DeaththreatXTime + PEmployXTime + MMSSS1XTime + MLDQXTime |  | intercepts | 363.8 | 402.9 | 325.8 | 19 | 16 | 232.2* |

*Note.* To obtain a parsimonious model during model building process, various predictors were removed based on assessment of p-values, pseudo-R^2^ values, and model information criteria (e.g., likelihood ratio tests). Only final models are depicted above. Abbreviations: Random effects (RE); Fixed effects (FE); Akaike’s Information Criterion (AIC); Schwarz’s Bayesian Criterion (BIC); -2 Log Likelihood (-2 LL); Likelihood ratio test (LRT); Maternal Living Difficulties Questionnaire (MLDQ); Paternal employment (1=Yes; PEmployment); Maternal MacArthur Subjective Social Status 1 (MMSSS1).

**Table S.2**

*Model comparison and model building process for anxiety*

| Sampling Units | | N total observations = 412  N subjects = 291 | | | | | | | | | |
| --- | --- | --- | --- | --- | --- | --- | --- | --- | --- | --- | --- |
|  | | | | | | | | | | | |
| Model Specification | **Model Name** | **Nested Model** | **Fixed Effects** |  | **Random Effects** | **Model fit** | | | | **LRT Test against nested** | |
|  |  |  |  |  |  | **AIC** | **BIC** | **-2 LL** | **df** | **df** | **X^2^** |
| RE only | Null | - | - |  | intercepts | 3353.3 | 3365.4 | 3347.3 | 3 | - | - |
| FE main effect | Model 1 | Null | Time |  | intercepts | 3323.1 | 3339.1 | 3315.1 | 4 | 1 | 32.2* |
| FE two-way interactions; controls | Model 2 | Model 1 | Time + Age + Sex + Victimization + Death Threat + Accident/Injury + AgeXTime + SexXTime + VictimizationXTime + Death ThreatXTime + Accident/InjuryXTime |  | intercepts | 2755.6 2837.6 2755.6 14 | | | | 10 | 591.7* |
| FE two-way interactions | Model 3 | Model 1 | Time + Age + Sex + Victimization + Death Threat + PEmployment + MPREQIs + PPREQIs + MMSSS1 + AgeXTime + SexXTime + VictimizationXTime + DeaththreatXTime + PEmployXTime + MMSSS1XTime + PPREQIsXTime + MPREQIsXTime |  | intercepts | 788.6 | 840.6 | 748.7 | 20 | 16 | 2566.4* |

*Note.* To obtain a parsimonious model during model building process, various predictors were removed based on assessment of p-values, pseudo-R^2^ values, and model information criteria (e.g., likelihood ratio tests). Only final models are depicted above. Abbreviations: Random effects (RE); Fixed effects (FE); Akaike’s Information Criterion (AIC); Schwarz’s Bayesian Criterion (BIC); -2 Log Likelihood (-2 LL); Likelihood ratio test (LRT); Maternal Living Difficulties Questionnaire (MLDQ); Paternal employment (1=Yes; PEmployment); Maternal Perceived Residential Environment Quality Indicators (MPREQIs); Paternal Perceived Residential Environment Quality Indicators (PPREQIs); Maternal MacArthur Subjective Social Status 1 (MMSSS1).

**Table S.3**

*Model comparison and model building process for depression*

| Sampling Units | | N total observations = 303  N subjects = 291 | | | | | | | | | |
| --- | --- | --- | --- | --- | --- | --- | --- | --- | --- | --- | --- |
|  | | | | | | | | | | | |
| Model Specification | **Model Name** | **Nested Model** | **Fixed Effects** |  | **Random Effects** | **Model fit** | | | | **LRT Test against nested** | |
|  |  |  |  |  |  | **AIC** | **BIC** | **-2 LL** | **df** | **df** | **X^2^** |
| RE only | Null | - | - |  | intercepts | 2275.3 | 2286.5 | 2269.3 | 3 | - | - |
| FE main effect | Model 1 | Null | Time |  | intercepts | 2248.4 | 2263.2 | 2240.4 | 4 | 1 | 28.9* |
| FE two-way interactions; controls | Model 2 | Model 1 | Time + Age + Sex + Victimization + Death Threat + Accident/Injury + AgeXTime + SexXTime + VictimizationXTime + Death ThreatXTime + Accident/InjuryXTime |  | intercepts | 2177.1 2228.7 2149.1 14 | | | | 10 | 91.3* |
| FE two-way interactions | Model 3 | Model 1 | Time + Age + Sex + Victimization + Death Threat + PPREQIs + PMSSS1 + AgeXTime + SexXTime + VictimizationXTime + DeaththreatXTime |  | intercepts | 500.5 | 531.8 | 472.5 | 14 | 10 | 1767.9* |

*Note.* To obtain a parsimonious model during model building process, various predictors were removed based on assessment of p-values, pseudo-R^2^ values, and model information criteria (e.g., likelihood ratio tests). Only final models are depicted above. Abbreviations: Random effects (RE); Fixed effects (FE); Akaike’s Information Criterion (AIC); Schwarz’s Bayesian Criterion (BIC); -2 Log Likelihood (-2 LL); Likelihood ratio test (LRT); Paternal Perceived Residential Environment Quality Indicators (PPREQIs); Paternal MacArthur Subjective Social Status 1 (PMSSS1).

**Percent Missing Data by Phase**

**Symptom Data**

Phase 1

SCARED: 42.9%

MFQ: 68.9%

UCLA PTSD RI: 36.7%

Phase 2

SCARED: 8.7%

MFQ: 12.8%

UCLA PTSD RI: 10.3%

Phase 3

SCARED: 4.3%

MFQ: 4.3%

UCLA PTSD RI: 4.3%

**Trauma Exposure**

Phase 1

LEC-5: 2.1%

Phase 2

LEC-5: 1.0%

Phase 3

LEC-5: 2.2%

**Demographics**

Phase 1

Age : 0.0%

Sex : 0.0%

Ethnicity : 0.0%

Country of origin: .6%

Phase 2

Age : 0.0%

Sex : 0.0%

Ethnicity : 33.1%

Country of origin: 31.3%

Phase 3

Age : 1.4%

Sex : 0.0%

Ethnicity : 33.1%

Country of origin: 33.8%

**Parental Predictors**

Phase 1

Maternal LDQ: 59.9%

Maternal Education: 3.4%

Maternal MacArthur SSS 1: 56.5%

Maternal MacArthur SSS 2: 56.5%

Maternal PREQIs: 79.1%

Paternal LDQ: 59.3%

Paternal Education: 22.0%

Paternal MacArthur SSS 1: 59.3%

Paternal MacArthur SSS 2: 40.7%

Paternal PREQIs: 81.9%

Phase 2

Maternal LDQ: 16.4%

Maternal Education: 45.6%

Maternal Employment: 10.3%

Maternal MacArthur SSS 1: 14.9%

Maternal MacArthur SSS 2: 14.9%

Maternal PREQIs: 52.3%

Paternal LDQ: 23.6%

Paternal Education: 50.3%

Paternal Employment: 50.8%

Paternal MacArthur SSS 1: 26.7%

Paternal MacArthur SSS 2: 26.7%

Paternal PREQIs: 38.5%

Phase 3

Maternal LDQ: 15.1%

Maternal Education: 54.7%

Maternal Employment: 56.8%

Maternal MacArthur SSS 1: 15.1%

Maternal MacArthur SSS 2: 15.1%

Maternal PREQIs: 48.9%

Paternal LDQ: 27.3%

Paternal Education: 58.3%

Paternal Employment: 59.0%

Paternal MacArthur SSS 1: 31.7%

Paternal MacArthur SSS 2: 31.7%

Paternal PREQIs: 58.3%

Note: We used data from the first follow-up visit post-arrival (Phase 2) for parental variables (MacArthur SSS 1 and 2, employment, LDQ, and PREQIs), as this time point provided a more realistic reflection of the resettlement environment. In contrast, data collected at arrival were likely too early to meaningfully capture the post-migration context. We also considered incorporating these variables as time-varying across all three waves. However, doing so introduced excessive model complexity and estimation burden in our linear mixed models without sufficient added value. Thus, Phase 2 data were selected to optimize both interpretability and model parsimony.
